# Amyloids in Site-Specific Autoimmune Reactions and Inflammatory Responses

**DOI:** 10.3389/fimmu.2019.02980

**Published:** 2020-01-09

**Authors:** Yan-Mei Huang, Xue-Zhi Hong, Jian Shen, Li-Jun Geng, Yan-Hong Pan, Wei Ling, Hai-Lu Zhao

**Affiliations:** ^1^Department of Immunology, Guangxi Area of Excellence, Guilin Medical University, Guilin, China; ^2^Center for Systems Medicine, Guangxi Key Laboratory of Excellence, Guilin Medical University, Guilin, China; ^3^Department of Rheumatology and Immunology, The First Affiliated Hospital of Guilin Medical University, Guilin, China; ^4^Department of Pathology, The First Affiliated Hospital of Guilin Medical University, Guilin, China; ^5^Department of Endocrinology, Xiangya Medical School, Central South University, Changsha, China; ^6^Institute of Basic Medical Sciences, Faculty of Basic Medicine, Guilin Medical University, Guilin, China

**Keywords:** amyloid, amyloid conformation, autoimmune, proinflammation, immunomodulation, homeostasis, Alzheimer's disease, diabetes

## Abstract

Amyloid deposition is a histological hallmark of common human disorders including Alzheimer's disease (AD) and type 2 diabetes. Although some reports highlight that amyloid fibrils might activate the innate immunity system via pattern recognition receptors, here, we provide multiple lines of evidence for the protection by site-specific amyloid protein analogs and fibrils against autoimmune attacks: (1) strategies targeting clearance of the AD-related brain amyloid plaque induce high risk of deadly autoimmune destructions in subjects with cognitive dysfunction; (2) administration of amyloidogenic peptides with either full length or core hexapeptide structure consistently ameliorates signs of experimental autoimmune encephalomyelitis; (3) experimental autoimmune encephalomyelitis is exacerbated following genetic deletion of amyloid precursor proteins; (4) absence of islet amyloid coexists with T-cell-mediated insulitis in autoimmune diabetes and autoimmune polyendocrine syndrome; (5) use of islet amyloid polypeptide agonists rather than antagonists improves diabetes care; and (6) common suppressive signaling pathways by regulatory T cells are activated in both local and systemic amyloidosis. These findings indicate dual modulation activity mediated by amyloid protein monomers, oligomers, and fibrils to maintain immune homeostasis. The protection from autoimmune destruction by amyloid proteins offers a novel therapeutic approach to regenerative medicine for common degenerative diseases.

## Introduction

Amyloids refer to misfolding protein aggregates which convert from their soluble physiological monomers under certain endogenous or exogenous conditions (1, 2). The amyloid aggregates include soluble non-fibrillar intermediates, such as α-helix-rich oligomers and protofibrils, and insoluble deposits of β-sheet fibrils. Ever since amyloid was initially used to describe the specific microscopic abnormalities during autopsy examinations in 1639, the protein deposition has been recognized as a hallmark of a variety of disorders with disparate symptoms (3–6). At present, nearly 50 disorders are associated with the formation of extracellular amyloid fibrils or intracellular amyloid-like confirmations (7). Diseases associated with formation of extracellular amyloid deposits are designated as amyloidosis (8).

The innate immune mechanism induced by amyloid deposition plays a remarkable role in the pathogenesis of amyloidosis (9). In Alzheimer's disease (AD), microglia and astrocytes can recognize deposition of amyloid β (Aβ) via specialized pattern recognition receptors (PRRs), such as toll-like receptors (TLRs), nucleotide-binding oligomerization domain-like receptors (NLRs), complements, receptor for advanced glycation end product, and scavenger receptors (10). Ligation of CD36, TLR2, TLR4, and TLR6 leads to proinflammatory signal transduction (11, 12). While the proinflammatory cytokines derived from activated cells initially account for the phagocytosis of Aβ, the self-sustaining proinflammatory mediators elicit inducible isoform of nitric oxide (NO) synthase and local NO production to induce neuronal apoptosis, axonal and synaptic damage, and inhibition of mitochondrial respiration (11). Likewise, the interaction between Aβ deposition and chronic inflammation contributes to the malfunction and death of neurons and eventually leads to AD development and progression. Consistent with the roles of Aβ plaques in AD, type 2 diabetes (T2DM)-related islet amyloid polypeptide (IAPP) deposits (13, 14) in pancreatic islets also can activate the pyrin domain-containing 3 (NLRP3) inflammasome and generate mature proinflammatory cytokine interleukin (IL)-1β (15), resulting in β-cell dysfunction and insulin deficiency (16, 17).

However, all immunotherapies targeting the clearance of amyloid under the assumption of the amyloid pathogenesis have proven unsatisfactory (18–22). In this framework of these treatments, immunotherapy has been historically regarded as a promising approach. For instance, active anti-Aβ immunization AN-1792 in phase IIA trial provides evidence of amyloid amelioration, but fatal subacute aseptic meningoencephalitis occurred in vaccine recipients. The meningoencephalitis is autoimmune-related due to autoreactive CD4+T cells infiltrating after autopsy examination. More intriguingly, injection of Aβ peptide into experimental autoimmune encephalomyelitis (EAE) delays disease onset; conversely, EAE gets worse following genetic deletion of the amyloid precursor protein (APP) (23). Other amyloid-forming proteins consistently exhibit such therapeutic outcome with highly immune suppressive activity (24). Such improvement in EAE conferred by Aβ treatment, to some extent, indicates the potential protective role of amyloid in defending against autoimmunity.

Hence, we summarize amyloid researches pertaining to the roles of amyloids in immune regulation with particular emphasis on the protective role of autoimmunity.

## Discovery and Evolution of Amyloid

The term amyloid was initially coined and popularized by Rudolph Virchow in 1854 to describe an abnormal change in the liver due to an iodine-staining reaction similar to that of starch. In fact, these “lardaceous” and “white stone” entities in other autopsy organs—consistent with the presence of amyloid—were described as early as in 1639 in autopsies by Nicolaus Fontanus. While there was no clear acknowledgment of the nature of amyloid between starch and cellulose until 1859, the absence of carbohydrate in a “mass” of amyloid and presence of highly proteinaceous species was established by Friedrich August Kekulé, who clarified that amyloid refers to a protein or a class of proteins (25).

Subsequent understanding of amyloid characteristics evolved with the development of more advanced techniques. The histochemical feature of binding Congo red with green birefringence was introduced by means of polarized light in the 1920s. In 1959, Cohen first identified characteristic elongated and unbranched fibrils, which differed from branching and thick collagen fibers under electron microscope (26). Furthermore, the biochemical feature of amyloid was identified in 1968 when Pras et al. solubilized and extracted insoluble amyloid fibrils (27). The two major types of breakdown subunits of amyloid fibrils were identified as amyloid light chain (AL) (28), derived from the light chain of an immunoglobulin and the other as amyloid A (AA) protein (29), which related to the primary and secondary form of the disease, respectively. Other common structures of amyloid fibrils include characteristic cross-β X-ray diffraction patterns, steric zippers of amyloid spine, and residues aligned in register (30–32).

## The States of Amyloid Proteins

### The Structures of Different Amyloid Protein States

Amyloid proteins mainly show three conformational structures: physiological monomers, pathological intermediates such α-helix-rich oligomers (e.g., dimers, trimers, dodecamers, and larger oligomers) and protofibrils, and eventually β-sheet amyloid fibrils. Intriguingly, amyloid fibrils with the common tinctorial properties and structural similarities are derived from non-homologous amyloid-forming proteins, which possess highly divergent sequence lists, secondary structure, and functions (Table 1). For example, some of the amyloidogenic proteins mainly display a high proportion of β-sheets; in other cases with α-helical and β-sheet mixture, even a rich α-helical structure is present. While during β-structure amyloid fibrils assembly, it has also been demonstrated that a similar kinetically intermediate state occurs—transient appearance of α-helical conformation (33–36). The phenomenon that physiologically disparate proteins from different sources convert to common structures of intermediates and fibrils remains an interesting mystery to explore. Furthermore, the terminal amyloid fibrils, beyond just the one core primary protein component, codeposit with common additional associated species, including metal ions, glycosaminoglycans, the serum amyloid P (SAP) component, apolipoprotein E, collagen, and many other minor components (2, 37, 38).

**Table 1:** Distinct structures of common amyloid proteins in clinical conditions.

**Systemic or localized**	**Pathophysiology**	**Aggregated protein**	**Amino acids**	**Molecular weight (kDa)**	**Structure/conformation of proteins**
Localized	Endocrine hormones	Amylin/IAPP	37	4	Natively disordered β-sheets
Localized	Endocrine hormones	Calcitonin	32	3.4	75% α-helical^*^
Localized	Endocrine hormones	Atrial natriuretic factor	28	3.1	β-turn and β-sheet mixed conformation
Localized	Endocrine hormones	Insulin	21+31	5.8	3 helices and the three disulfide bridges
Localized	Endocrine hormones	Prolactin	199	23	Four major a helixes with two antiparallel pairs
Systemic	Transport molecules	Transthyretin	127	15	β-sheet-rich content and one short α-helical
Systemic	Immunity/inflammation	β2-Microglobulin	99	11	β-pleated sheet
Systemic	Immunity/inflammation	Cystatin C, variants	120	13.3	Mainly antiparallel β-sheets
Systemic	Immunity/inflammation	Lysozyme, variants	130	14.3	42% α-helical and 4% β-sheet^*^
Systemic	Immunity/inflammation	Fibrinogen α, variants	27~136^#^	3~12	68% α-helical^*^
Systemic/Localized	Immunity/inflammation	Immunoglobulin light chain or fragment	~90^#^	~12	Antiparallel β-sheet
Systemic/Localized	Immunity/inflammation	Immunoglobulin heavy chain fragment	52~228^#^	6–22	Antiparallel β-sheet
Systemic	Immunity/inflammation	Serum amyloid A fragment	45~104^#^	4.5~11.5	Antiparallel four helical bundle structure
Systemic	Transport molecules	Apolipoprotein A I fragment	80~93^#^	8.9~10.8	High content of anti-parallel amphipathic α-helical
Systemic	Transport molecules	Apolipoprotein A II fragment	98	10	α-helical
Systemic	Transport molecules	Apolipoprotein A IV fragment	~70^#^	~8	α-helical
Localized	Transport molecules	Lactoferrin	692	82.4	36% α-helical and 15% β-sheet^*^
Localized	Nervous system	α-Synuclein	140	14.5	59% α-helical^*^
Localized	Nervous system	Tau	352–441	36.8~45.9	Natively disordered microtubes
Localized	Nervous system	Amyloid-β peptide	40 or 42	4.3~4.5	Aβ40: cross-β; Aβ42: β-sheet (in aqueous buffers)
Systemic	Nervous system	Prion protein (PrP^sc^) or fragment	253	27.6	High proportion of β-sheet structure
Systemic	Cell motility	Gelsolin, variant	71	8	Five-stranded β-sheet, flanked by two α helices
Systemic	Cell cycle or repair	Leukocyte chemotactic factor-2	133	15	8% α-helical and 29% β-sheet (Chain A within two chains)^*^
Localized	Cell growth control	Galectin 7	136	15	49% β-sheet (Chain A within two chains)^*^
Localized	Lung function	Lung surfactant protein C	35	4	11% α-helical and 19% β-sheet (Chain A within six chains)^*^

* *Secondary structure derived from the Protein Data Bank (PDB)*.

# *Fragments of various lengths are reported in ex vivo fibrils*.

### The Role of the Amyloid States in Diseases

Distinct native proteins in humans serve different biological functions *in vivo*, such as endocrine hormones, transport molecules, immunity response, and normal cell function control. Usually, protein aggregation has unique biophysical characteristics with dramatical impact on cell activity. The smaller species of amyloids such as oligomers and protofibrils are considered to be the most toxic form in the process of membrane perforation and cell degeneration.

Soluble Aβ oligomers have been shown to induce memory deficits and cognitive impairment in transgenic mice (39, 40). In addition, these purified soluble assemblies from brains of impaired Tg2576 mice disrupt memory when administered to young rats. The reduction in oligomer levels, on the other hand, corresponded to improved memory in these mice (41). Tg2576 mice with amyloid plaques did not show memory function and behavioral deficits during episodes of markedly reduced levels of Aβ oligomers. Moreover, these soluble oligomers were also toxic to cultured neuronal cells by inducing membrane depolarization (42–44). Similarly, IAPP oligomers induce insulin-producing β cell mass loss and apoptosis *in vitro* cell cultures and in transgenic mice (45, 46), consistent with the findings of oligomer-specific immunoreactivity and β cell depletion in islet cells of patients with T2DM (47). Such cell toxicity exhibited by oligomeric species have also been seen in other amyloid-related disorders including Parkinson's disease (α-synuclein), spongiform encephalopathies [prion protein (PrP)], Huntington's disease, and spinocerebellar ataxias (polyQ proteins) (43).

The toxicity of oligomers is not specific, and they interact with many targets, including membrane disruption interaction, mitochondrial dysfunction, oxidative stress, and reactive oxygen species production, suggesting that toxicity is associated with the formation process rather than a specific oligomeric species. It is generally assumed that toxicities of oligomers of different proteins are mediated by a common sequence-independent conformation, implying a common mechanism of pathogenesis of all the amyloidoses (48–50).

Instead of acting as an etiological agent, amyloid fibrils have three major disparate roles as described for differential amyloid deposition according to growing evidence (Table 2). First, the formation of amyloid fibrils does not necessarily denote causality with diseases for the following reasons: (1) weak correlation between Aβ deposits and cognitive status (18, 53, 54); (2) lack of correlation between loss neural function within the regions responsible for memory and the extent of Aβ deposits in that brain region (55–58); (3) oxidative stress precedes fibrillar depositions of Aβ (59–61); (4) amyloid fibrils are the product of the innate immune response (62–64); (5) Aβ plaques were identified in cognitively normal elderly people (65–67); (6) animals with Aβ deposition do not develop the clinical signs of the cognitive impairment (68); and (7) treatments targeting on Aβ plaques have been unsuccessful. Second, biophysical or functional amyloids have been described broadly from bacteria to humans (95–97), such as curli biogenesis (69–72), silkmoth chorion generation (73), melanin and other hormones synthesis (74, 75), epigenetic control of polyamines (76, 77), and other biological functions (78–81). Furthermore, to a far greater extent than anyone suspected, amyloid wields potential for protective roles (98), including neuroprotective action (82–85), defending against oxidative damage (86–89), prion (90, 91), and metal-induced toxicity (92–94), and protecting against microbial infection and autoimmune destruction.

**Table 2:** Specific actions of amyloid protein and deposition denoted in research evidence.

**Actions**	**Evidence**	**Representative reference number**
Macroscopic abnormalities	Lardaceous changes in liver, spleen, heart, islets, and kidneys	(3, 51)
Aetiological agent	Alzheimer's disease: amyloid cascade hypothesis	(52)
	Type 2 diabetes	(13, 14)
Product rather than the cause, secondary to other pathogenic events	Alzheimer's disease	
	Weak correlation between Aβ deposits and cognitive status	(18, 53, 54)
	Lack of correlation between loss neural function within the regions responsible for memory and the extent of Aβ deposits in that brain region	(55–58)
	Oxidative stress precedes fibrillar depositions of Aβ	(59–61)
	Amyloid fibrils are the product of the innate immune response	(62–64)
	Aβ plaques were identified in cognitively normal elderly people	(65–67)
	Animals with Aβ deposition do not develop clinical signs of the cognitive impairment	(68)
	Treatments targeting on the Aβ plaques have been unsuccessful	(18–22)
Functional amyloid/biological function	Bacterial and mammalian systems	
	Curli and aerial hyphae biogenesis	(69–72)
	Silkmoth chorion generation	(73)
	Melanin and other hormones synthesis	(74, 75)
	Epigenetic control of polyamines	(76, 77)
	Haemostatic role	(78)
	Molecular memory	(79, 80)
	Information transfer	(81)
Protective roles	Neuroprotection (Aβ)	(82–85)
	Antioxidant (Aβ and tau)	(86–89)
	Inhibit Aβ toxicity (tau); inhibit prion toxicity	(90, 91)
	Protect against metal-induced toxicity	(92–94)
	Defend against autoimmunity	23 (Aβ42 and Aβ40) 24 (amyloid fibrils) 138 (amyloid fibrils composed of hexameric peptides) 141 (amyloid-forming peptides that exhibited chaperone activity) 139 (mechanism) 140 (mechanism) (51)
	Anti-microbial (Aβ, IAPP and a-synuclein)	147 (Aβ) 150 (Aβ and aggregated Aβ) 151 (IAPP) 152 (α-Syn)

## Diseases Featuring Amyloid

Different amyloid-forming proteins are associated with different diseases. According to the International Society of Amyloidosis, there are 36 known extracellular amyloid fibril proteins associated with amyloidoses in humans, 2 of which are iatrogenic in nature and 9 of which have also been identified in animals (8).

The amyloidoses are classified as systemic or localized forms based on location and extent of amyloid protein buildup. Three common conditions associated with systemic amyloidosis are primary amyloidosis (also called AL), familial (hereditary) amyloidosis, and secondary amyloidosis (AA amyloidosis) such as tuberculosis or rheumatoid arthritis. Secondary amyloidosis, characterized by the deposition of serum amyloid A (SAA), occurs as a complication of an existing chronic infection or chronic inflammatory disease. Infections and inflammation stimulate human liver to produce high levels of SAA. With ongoing inflammation, a small portion of the SAA protein, called AA protein, might separate from SAA and deposit in tissues as AA amyloid. Although the mechanism of partial breakdown of SAA to AA is not well-understood, tuberculosis with chronic states of inflammation usually induces renal complications of SAA-induced amyloidosis. Besides, β2-microglobulin amyloidosis affects people who undergo long-term hemodialysis or continuous ambulatory peritoneal dialysis. T2DM and AD represent two common clinical conditions of localized amyloidosis. Another study broadly groups amyloidoses into neurodegenerative conditions, non-neuropathic localized amyloidosis, and non-neuropathic systemic amyloidosis (2). The chemical characterization of the precursor protein is also recommended as an approved classification criterion (8).

Some hypotheses have been put forward for the pathogenesis of amyloidoses, in which normally soluble proteins aggregate into regular, insoluble amyloid fibrils. Any methods that enhance the sources of amyloidogenic peptide and/or decrease clearance of amyloid-forming proteins contribute to amyloid formation and lead to amyloidoses eventually (99). Genetic variability of amyloidogenic protein as a negative factor leads to a vulnerable process of amyloid assembly. For instance, amyloid transthyretin amyloidosis is caused by mutations in the TTR gene (100, 101). Cleavage of the precursor protein contributes to an overproduction of the amyloidogenic protein and enhances the propensity for encouraging amyloid fibrils formation. One typical instance is that Alzheimer-related Aβ, which is cleaved by β-secretase and γ-secretase (102). It is also theoretically recognized that the “nucleated growth” mechanism is one of the characteristics of amyloid fibril formation. Once lag phase “nuclei” form, mature fibrils grow rapidly by association with various oligomers or monomers (2). In addition, intrinsic properties—referring to amino acid sequences and conformation states—and external conditions—such as pH, temperature and pressure—both serve as important factors to the susceptibility of a given polypeptide chain to convert into amyloid fibrils (103).

## Amyloid as a Proinflammatory Factor

### Amyloid-β and Proinflammation

**Figure 1:**
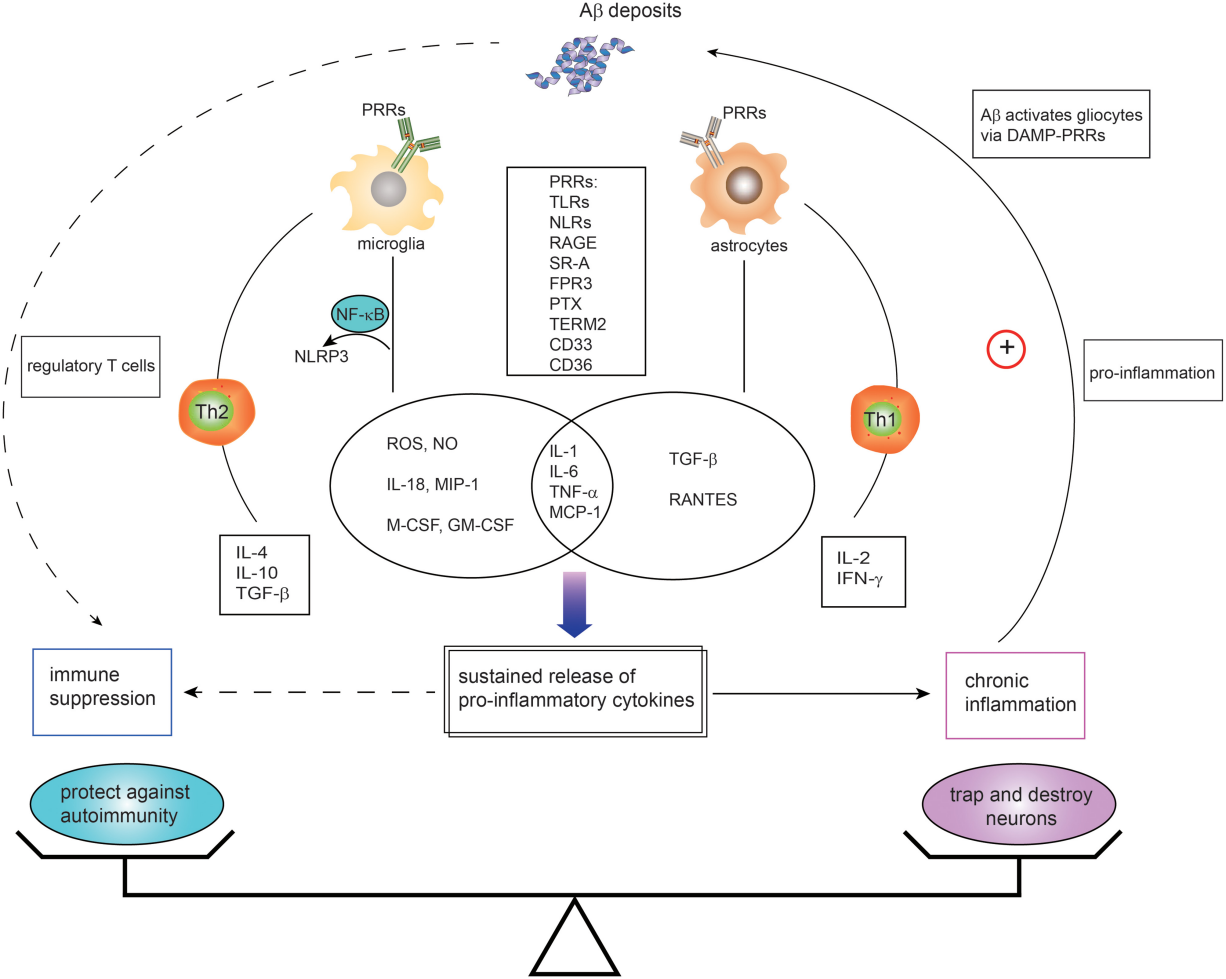
Dual immunomodulation of brain amyloid β deposits in the context of Alzheimer's disease. Microglia and astrocytes, expressing innate immune receptors—pattern recognition receptors (PRRs)—can be activated in response to amyloid-β (Aβ) via DAMPs-PRRs ligation. Such PRRs include toll-like receptors (TLRs), nucleotide-binding oligomerization domain-like receptors (NLRs), receptor for advanced glycation end products (RAGE), scavenger receptors (SRs), N-formyl peptide receptors (FPRs), pentraxin (PTX), triggering receptor expressed by myeloid cells 2 (TREM2), CD36, and CD33. The activation of microglia and astrocytes leads to secretion of proinflammatory cytokines and chemokines. Activated microglia with TLRs agonist or cytokines also activate NALP3 inflammasomes via nuclear factor kappa B (NF-κB) mediated signaling, resulting in production of proinflammatory mediators. While the proinflammatory cytokines derived from activated cells initially account for the phagocytosis of Aβ deposition, the self-sustaining proinflammatory mediators and chronic inflammation contribute to the malfunction and death of neurons and eventually leads to AD development. Likewise, the chronic neuroinflammation in turn hastens cycle reinforcing Aβ deposition. In addition, Th1 cells and Th2-polarized cells are activated with implication in proinflammatory and anti-inflammatory regulation. However, direct and indirect evidence is emerging that Aβ has immune suppressive activity and protects against autoimmune disorders. It is likely that the states and activities of brain Aβ might be orchestrated by a whole variety of different factors. TNF, tumor necrosis factor; TGF, transforming growth factor; IFN, interferon; DAMPs, damage-associated molecular patterns; TREM2, triggering receptor expressed by myeloid cells 2; IL, interleukin. G-CSF, granulocyte colony-stimulating factor; GM-CSF, granulocyte macrophage colony-stimulating factor; IFN-γ, interferon-gamma; M-CSF, macrophage colony stimulating factor; NLRP3, NOD-like receptor, type 3; NO, nitric oxide; ROS, reactive oxygen species; SRs, scavenger receptors; TLRs, Toll-like receptors; MIP-1, macrophage inflammatory protein 1; MCP-1, monocyte chemoattractant protein-1; RANTES, regulated on activation, normal T cell expressed and secreted.

Extracellular deposits of Aβ protein forming senile plaques represents pathognomonic for AD. The triggering of immune reactions leads to inflammatory processes in the brain (Figure 1). The most influential model has been the “amyloid cascade hypothesis” proposed by Hardy nearly 30 years ago (52). The innate immune system, as the first line of defense against pathogen-associated molecular patterns or damage-associated molecular patterns, is triggered in response to sensing emerging Aβ plaques via the PRRs expressed in microglia and astrocytes. Activated microglia play an indispensable role in surveying the brain milieu by producing the proinflammatory cytokines IL-1, IL-6, and tumor necrosis factor alpha (TNF-α), and chemokines IL-8, macrophage inflammatory protein-1, monocyte chemoattractant peptide-1, the growth factor macrophage-colony stimulating factor and complement cascade proteins (104, 105). Reactive astrocytes also can secrete proinflammatory mediators such as monocyte chemoattractant peptide-1 (MCP-1), regulated on activation, normal T cell expressed and secreted (RANTES), TNF-α, and IL-1 in response to stimulation with Aβ42 (106). A wide array of PRRs in glial cells and neurons are implicated in Aβ hypothesis cascade, such as TLRs, NLRs, receptor for advanced glycation end products, complements, and scavenger receptors (11, 12, 107).

In addition, insoluble fibrillar Aβ can activate the NALP3 inflammasome dependent on lysosomal damage in mouse brain phagocytic cells (108, 109), which results in the release of IL-1β and cathepsin B followed by caspase-1 activation and secretion of several proinflammatory and chemotactic mediators (110). Plasmacytoid dendritic cells as another innate immune cell population are potently activated by nucleic-acid-containing amyloid fibrils (111).

The activated signaling pathways in the early stage of AD play a protective role by phagocytosis and degradation of amyloid aggregates (9, 112). However, along with continued production of the amyloid clumps, chronic stimulation of the innate immune system leads to persistent inflammation. Such chronic inflammation results in serious disadvantages, such as immune-competent cells deprivation, more APP synthesis, and cellular dysfunction or death (113, 114). This is the dichotomous role of amyloid-induced inflammation. Therefore, since amyloid plaque usually present with chronic inflammation, a plethora of studies have generally connected amyloid with the pathogenesis process of AD (115–117).

### Islet Amyloid Polypeptide and Proinflammation

Consistent with the role of Aβ in AD, T2DM-related IAPP deposits in islets of the pancreas can activate the NLRP3 inflammasome and generate mature proinflammatory cytokine IL-1β in transgenic mice model (15). The proposed mechanism underlying the release of proinflammatory cytokine is attributed to intraislet macrophages with increased expression of cell surface Ly6C and CD11c as induced by IAPP aggregates (118). In macrophage-prime cultured medium, IL-1β secretion is TLR2 dependent or NLRP3 dependent based on distinct IAPP aggregation states (119). Islet β cells are also a potential source of proinflammatory cytokines, which are associated with β-cell dysfunction in patients with T2DM (16, 17).

Indeed, other amyloidogenic peptides, including SAA (120, 121), show common activation pathways that trigger an innate immune response via binding multiple cell surface PRRs comprised of RIG-I-like receptor, NLR and TLR.

## Amyloid as an Immunosuppressive Factor

### Amyloid β in Immunomodulation

The common immunosuppressive regulation signaling pathways include stimulation of cytokines production and several TLRs, as well as lipopolysaccharide-induced tolerance of innate immunity system. The overexpressed pleiotropic cytokines IL-10, IL-6, and IL-1 (122–126) and increased CD33 expression (127, 128) within affected cerebral cortical regions of AD have anti-inflammatory and immunosuppressive properties. Variant triggering receptors expressed on myeloid cells 2 binding tyrosine kinase-binding protein expressed by microglia cells downregulate inflammatory signaling in response to TLR ligation (129, 130). Hickman et al. also reported that the inflammatory cascade initiator of receptor CD36 in microglia for Aβ binding displayed a major decrease in the AD transgenic mice (131). In addition, suppressors of cytokine signaling highly expressed in the astrocytes and microglia of AD patients are indicative of cytokine-JAK-STAT pathway inhibition, as well as adaptive and innate immune response downregulation (132, 133).

### Amyloid-Ameliorated Experimental Autoimmune Encephalomyelitis

To explore the amyloid deposition mechanism that underlies multiple sclerosis-like brain inflammation known as EAE, in 2012, Steinman et al. injected synthetic Aβ40 or Aβ42 peptides peripherally into four different models of EAE (23). Unexpectedly, Aβ could neither cross the blood–brain barrier nor stimulate immune cells to attack neurons, whereas it relieved symptoms associated with EAE. To further explain the mechanism underlying Aβ-mediated suppression of EAE, the researchers measured more than 20 cytokines and chemokines in the blood of Aβ-treated mice and found overall dampening of proinflammatory signaling molecules, most notably IL-6, IL-2, transforming growth factor-β and IL-17, as well as key components of the well-known EAE-pathogenetic Th1 and Th17 pathways. In the periphery, treated mice exhibited an increased subset of lymphocytes effector T cells (CD4+CD62L+) that produced higher amounts of interferon-gamma (IFN-γ), which acted to suppress the immune system in EAE. Flow cytometry of immune cell subsets (T cells: CD4+ T cells, CD8+ T cells, CD4+CD62L+IFNγ + T cells, B cells: B1 and B2 subsets, and myeloid cells: neutrophils, mast cells, large peripheral macrophages, and small peripheral macrophages) revealed significant changes in the distribution and subtle expansion/depletion of immune subsets in the peritoneal cavity. Furthermore, in both mouse and human *in vitro* experiments, Aβ directly suppressed thymidine incorporation of activated EAE-specific CD4+ T cells. These *in vitro* experiments demonstrate that activated mouse and human CD4+ T cells are direct targets of Aβ immunosuppression. Thus, the collection of findings means that Aβ-induced amelioration of EAE may be related to production of immune suppressive cytokines, modulation of lymph node and myeloid compartments, and reduction in serum levels of proinflammatory signaling molecules.

Moreover, exacerbated clinical EAE occurs in mice with the genetic deletion of the AβPP (23). “Loss of function” experiments consolidate the observation with gene deletion of other amyloid-forming related molecules of PrP (134), tau (135), SAP (136), and molecular chaperone αB crystallin (Cryab). Subsequently, a core hexapeptide structure in amyloid-forming molecules was identified from a variety of notorious amyloid proteins including PrP, tau, Aβ, amylin, SAA, insulin, and molecular chaperone Cryab. Congruent with EAE protection, when these hexapeptides (Table 3) were given to engineered EAE mice with early clinical symptoms, they showed remarkable and consistent autoimmune therapeutic results (24). In addition, we have found that human IAPP could also induce CD4+CD25+Foxp3+ regulatory T cells and reduce the risk of autoimmune diabetes (51, 137). These findings collectively suggest localized autoimmune protection conferred by some kinds of amyloid forming proteins. More recent studies propose the benefits arising from several pathways of immune suppression, such as activating B-1a lymphocytes and F4/80+ macrophages, inducing a set of immune-suppressive cell-surface proteins (BTLA, IRF4, and Siglec G) and IL-10 (138). Two independent pathways are stimulated by amyloid fibrils, reduction in IL-6, TNF-α, and IFN-γ levels, and induction of type 1 IFN by plasmacytoid dendritic cells. These pathways act in concert to be immunosuppressive in Th1 indications (139, 140). Molecular chaperone activity and the capacity of binding proinflammatory proteins at the elevated temperatures within inflammatory foci are also correlated with the therapeutic activity of amyloid fibrils (24, 141). In addition, amyloid hexapeptide treatments improve the clinical outcomes of C57BL/6J (B6), but not B10 mice, indicating that genetic background might influence therapeutic efficacy (142).

**Table 3:** Hexapeptide-containing amyloid fibrils with therapeutic potentials for experimental autoimmune encephalomyelitis.

**Hexameric peptides**	**Amino acid**
HspB5 76–81	Ac S V N L D V CONH2
Insulin B chain 11–16	Ac V E A L Y L CONH2
Insulin A chain 12–17	Ac L Y Q L E N CONH2
HspB5 89–94	Ac L K V K V L CONH2
Aβ A4 protein 27–32	Ac N K G A I I CONH2
Tau 623–628	Ac V Q I V Y K CONH2
Serum amyloid P 213–218	Ac G Y V I I K CONH2
Aβ A4 protein 16–21	Ac K L V F F A CONH2
Major prion protein 148–153	Ac S N Q N N F CONH2
Apolipoprotein E 53–58	Ac S S Q V T Q CONH2
Amylin 28–33	Ac S S T N V G CONH2
Ig k chain 5–10	Ac S V S S S Y CONH2
Aβ A4 protein 29–34	Ac G A I I G L CONH2
Aβ A4 protein 35–40	Ac M V G G V V CONH2
Aβ A4 protein 37–42	Ac G G V V I A CONH2
Amylin 24–29	Ac G A I L S S CONH2

Indeed, as early as 2007, Steinman et al. found Cryab can attenuate inflammation in several models of inflammation, including ongoing EAE, downregulate antigen-specific Th1 and Th17 responses, and impact key inflammatory pathways such as nuclear factor kappa B and p38MAPK (143, 144). As these results conflict with van Noort's findings of multiple sclerosis in 1995 (145), the hint of amyloid-forming peptides' therapeutic potential has not yet come into consideration. It was Tanaka et al. who reported that the protein worked as a chaperone when it formed amyloid fibrils (146) that the unexpected idea that the molecule might be functioning in an amyloid state first dawned on various research fields.

### Amyloid-Ameliorated Inflammation

*In vitro*, synthetic Aβ exhibits potent antimicrobial activity toward microbial pathogens, fungi (147), and viruses (148, 149). The biological relevance of this protection by *in vitro* Aβ activities was validated in transgenic mice overexpressing human Aβ, demonstrating defense against meningitis. Intriguingly, Aβ aggregates entrap and imprison these pathogens (150). Particularly germane to Aβ's role in anti-inflammation, several other amyloidogenic peptides including IAPP (151), α-synuclein (152), serum amyloid A, microcin E492, temporins, and protegrin-1 also show antimicrobial properties (153).

In addition, Moir et al. propose that Aβ belongs to a family of antimicrobial proteins (AMPs) based on characteristics that Aβ shares with an AMP called LL-37 (147). Intriguingly, LL-37 plays the dual role of rousing inflammation in some scenarios and squelching it in others (154, 155). In this regard, the discovery implies that Aβ deposition appears to exert action in immune regulation. On the other hand, biophysical analysis showed an extensive β-sheet structure present in various species of bacterial fimbriae, suggesting that amyloids share common structural components of the extracellular matrix in bacterial biofilms (71, 156, 157). The established biofilms are stable communities and function as antimicrobials agents generally resistant to harsh denaturing. In addition, biofilms from both commensal and pathogenic microbiota recognized by TLR2 resulted in the production of an immunomodulatory cytokine of intestinal homeostasis that ameliorate inflammation in a mouse model of colitis (155, 158, 159). The amyloid fibrils with structural and functional similarity to the protective shell of bacterial biofilm implies that amyloid fibril structure exhibits beneficial behaviors in defending against autoimmune attacks.

### Amyloid β and Regulatory T Cells

The CD4+CD25+Foxp3+ regulatory T cells (Tregs) play a critical role in modulating the balance between inflammation and immune tolerance to prevent autoimmune diseases (160). In peripheral lymphocytes, the frequency and suppressive activity of Tregs are increased in patients with AD as compared to non-demented controls (161). Additional evidence reinforces the significant augmentation of the strongest suppressive subset of PD1-Tregs subpopulation in patients with mild cognitive impairment; however, this augmentation is lost in patients with severe AD (162). Previous review has revealed that Tregs non-specifically inhibit CD4+ T cell proliferation both *in vitro* and in EAE (163). It is worth noting, according to the Dorothee group, that Tregs critically control the magnitude of CD4+ T cell targeting to amyloid-β in response to amyloid-β vaccination in both physiological and pathological settings (164). Consistently, transient depletion of Tregs in mouse models of AD accelerates the onset of cognitive deficits; conversely, amplification of Tregs improves cognitive functions (165). Therefore, increased frequency and suppressive activities of Tregs within AD patients suggest a protective role in autoimmune disorders (166, 167).

### Amyloid and Adaptive Immunity

In contrast to intensive exploration of innate immunity in amyloid diseases, there has been comparatively limited research focusing on the impact of the adaptive immune system, in which self-reactive T cells or autoantibodies underlie autoimmune diseases.

In the 1970s, studies demonstrated that common amyloid-inducing agents were polyclonal B-cell activators *in vitro* as well as *in vivo* (168, 169). The role of adaptive immunity on AD pathogenesis remains ambiguous. Samuel et al. revealed dramatically increased Aβ plaque load in the “Rag-5xfAD” mice model, which lacked an adaptive immune response. Furthermore, genetic depletion of immunoglobulin G producing B cells exacerbated amyloid deposition, and this effect would be conversely ameliorated when again given immunoglobulin G through direct injection or bone marrow transplantation (170). Another study showed contradictory results that established PSAPP mice model of AD lacking functional B and T cells revealed reduced brain Aβ-peptide levels (171). The favorable and negative roles of the adaptive immune system, at least, suggest an impact of the adaptive immunity on cerebral β-amyloid pathology.

However, in view of the evidence from clinical trials applying Aβ-immunized vaccine showing the occurrence of specific CD4+ T cells targeting Aβ, we pay close attention to the changes of CD4+T cells in the brain, cerebrospinal fluid (CSF), and perivascular blood.

In several studies of the brains of patients with AD, CD4+ T cells have on occasion been found in the brain. In these cases, these CD4+ T cells have mainly been situated in the hippocampus and cortical regions of the brain and have only rarely appeared to colocalize with parenchymal Aβ deposits (172, 173). Similarly, the increased T cell in leptomeningeal and cortical vessels parallel with Aβ deposits indicate little evidence of intraparenchymal Aβ plaque driving T-cell infiltration into the AD brain (174). One study described a majority of T cells as existing in the parenchyma of AD patients; however, these T cells were memory cells expressing CD45RO+ and with the absence of proliferating nuclear proteins (i.e., Ki67 and PCNA) (172). A recent investigation further examined the activation state of T cells in transgenic mice models of AD-like cerebral amyloidosis and revealed hypo-responsiveness of the adaptive T cells response to Aβ, including the weak activation and proliferation, reduced expression of the effector cytokine IFN-γ, and downregulation of antigen presentation (175), which was in line with the concept of Aβ-induced immune hyporesponsiveness as early as in 2001 (176). Those findings, taken together, illustrate that the appearance of amyloid accompanied with regional declined/anergized disease-producing T-cell activation may imply a suppressive role of Aβ on localized autoimmune response.

In contrast to pathological brain-related T cells, T cells retained in the blood–CSF barrier of the choroid plexus usually mediate physiological immune surveillance. As such, in the research on T cells in CSF and T cells in peripheral circulating lymphocytes, there exist conflicting results.

One study pointed out that in circulating blood, there were no appreciable differences in the percentage of CD4+T cells between AD patients in different stages of dementia and the health control (177), whereas another study revealed that CD4+T lymphocytes exhibited a significant decline in the CSF as Aβ deposits in brain increased (178). However, several researches have illustrated that in both CSF and peripheral blood of mild AD patients, the proportion of activated CD4+ T cells was enhanced in comparison with age-matched, elderly controls (179, 180). The human leukocyte antigen DR isotype independent Aβ-reactive CD4+ T cells were also increased, while the magnitude of T-cell response showed great variable in different stages of dementia (181). With enhanced functioning of effector T cells, AD progression slows down (182). Whereas, as the severity of AD progressed, the number of CD4+T cells showed significant reduction in comparison with mild to moderate symptoms and controls (183). The interaction that exists between perivascular T subsets and Aβ pathological autoreactive T cells in the brain remains perplexing (184); however, the findings above may supply the beneficial role of Aβ and concomitant increased surveillance of circulating T cells.

## Amyloid and Autoimmunity

### Clearance of Amyloid-β Plaque Provoked Autoimmune Disorders

Since the notion of amyloid causality has become a dogma within the research field, the passion for removing amyloid fibrils has gone unabated.

Dating back to 2001, AN-1792 reached clinical trials as the first active immunotherapy targeting β-amyloid clearance. The preclinical results from the vaccine were promising. However, the phase IIA trial was halted completely in 2002 due to subacute aseptic meningoencephalitis that developed in 6% (18 of 298) vaccine recipients (185, 186). Neuropathological examinations of brain tissues showed clearance of amyloid plaques while notably accompanied with elevated levels of CD4+ T cells in perivascular distribution. Depletion of brain Aβ with T-cell activation was also noted in another post-mortem examination of a patient who participated in the phase I study (187). These autoreactive CD4+ T cells that infiltrate the brain can induce autoimmune reactions and are thought to be responsible for the presence of encephalitis (188). A potential mechanism that underlies post-vaccination meningoencephalitis currently remains debated. Adjuvant (QS-21)-related TH1 lymphocytes activation and presence of T-cell recognition epitopes (within amino acids 15–42 of Aβ) (186) in the Aβ peptide have been proposed to initiate the T responses that triggered the autoimmune meningoencephalitis in some AN1792-vaccinated patients (189).

We previously summarized frequent occurrence of another autoimmune response against cerebral amyloid angiopathy—amyloid-related imaging abnormities (ARIA)—in all the clinical trials using amyloid-centric agents (21). Alarmingly, 20 times higher risk of ARIA occurred in patients with reduced brain amyloid burden than those without amyloid plaque clearance. Moreover, ARIA often colocalized at sites with the highest level of amyloid reduction.

We propose that the side effect of active anti-Aβ immunization rarely derives from vaccine adjuvant or any other reasons. The presence of autoimmune-related side effects in the absence of Aβ deposition raises the concern of amyloid plaque's potential role in retarding or inhibiting immune responses of an organism against its own healthy tissue. The overlap of the appearance of inflammatory signs with the time of microglial activation after the Aβ immunization provides an explanation that Aβ clearance from the brain are concomitant with periods of inflammation (185). Thus, the removal of amyloid plaques may prevent it from serving its natural protective role (22) and lead to an autoimmunity impairment of brain function.

### Increased Prevalence of Autoimmunity Disorders in the Absence of Serum Amyloid P

SAP component is confirmed as a minor constituent binding to specific determinants shared by all types of amyloid fibrils (190). The concentration of SAP has been shown to be positively associated with amyloid deposits both *in vivo* and *in vitro* (191).

In many autoimmune disorders, improper clearance of nuclear debris released by apoptotic and necrotic cells is a potential source of autoantigens (192–195). Studies in mice with targeted deletion of the SAP gene spontaneously developed significant levels of antinuclear autoantibodies and autoimmune disease (191, 196). In addition, supplement of SAP into mice with systemic lupus erythematosus could effectively alleviate the autoimmune disease (197). The underlying mechanism indicates that SAP participates in handling of non-immunogenic apoptosis through reacting with nuclear autoantigens and inhibiting the formation of pathogenic autoantibodies targeted in systemic autoimmunity (198, 199).

Therefore, amyloid binding with SAP may potentially imply an important physiological role with regards to defense against autoimmune disorders.

### Presence of Autoimmune Diseases in the Absence of Islet Amyloid Deposition

Amylin, or IAPP, a 37-amino-acid peptide with amyloidogenic properties, is synthesized and cosecreted with insulin from pancreatic β cells and plays a critical role in modulating peripheral glucose balance. The deposition of islet amyloid is paralleled by progressive β-cell dysfunction found in 40–90% patients with type 2 diabetes (200, 201).

Type 1 diabetes (T1DM) is a form of autoimmune diabetes in which not enough amylin is produced. Then, the absence of islet amyloid deposition in patients with T1DM results in a greater risk of many autoimmune disorders, both organ and non-organ specific: thyroid disease, Addison's disease, coeliac disease, rheumatic disease, among others (202, 203). Similar latent autoimmune diabetes in adults (LADA) occurs with damaged β cells in the pancreas with inadequate amylin production. Various circulating autoantibodies including gliadin, endomysial, and thyroid antibodies are reported more frequently in patients with LADA than in T2DM patients (204).

Common histocompatibility antigens shared in both diseases that may explain autoimmune diseases often appear in clusters. Owing to islet amyloid formation not found in all T2DM patients and a limited number of studies, we cannot directly find more evidence that patients with IAPP deposition defend against localized or systematic autoimmune diseases. However, the coexistence of autoantibodies or autoimmune diseases present in T1DM and LADA broaden the view that lack of specific amyloid protein fibrils has some bearing on the presence of these autoimmune disorders.

## Concluding Remarks

The prevailing idea casting insoluble amyloid fibrils as strictly harmful has dominated the scientific literature for many centuries. However, based on the pathogenetic activation of proinflammatory states, anti-inflammatory strategies do not obtain any clinically satisfactory results. Moreover, autoimmune diseases that occur accompany with clearance of amyloid. Such results, along with the presence of toxic oligomers in pathogenesis of amyloidosis, represent a paradigm shift in our understanding of amyloid. An unconventional, new view is emerging in which some amyloid-forming proteins have a potential for doing good—far more so than anyone suspected; furthermore, their benefit is not just limited to antioxidant defense or an antibacterial function but has also been shown to exhibit therapeutic effects on autoimmune disorders, according to the latest evidence. The protective function from localized and systematic autoimmune disorders should be distinguished as exemplified in Aβ and IAPP. Indeed, amyloid with robust ordered β-sheet conformation resembles an armor, which to some extent traps and constrains cell dysfunctions while possessing a common protective role in different sites such as defensive biofilm in bacteria, antimicrobial peptide of IAPP and Aβ, and in our study, defending the body against immune attacks.

In summary, these arguments suggest that immune regulation mediated by amyloid plays a critical role in maintaining homeostasis between stimulating inflammation and defending against autoimmune responses. The appearance of amyloid represents a self-protective physiological phenomenon whereby the body engages in an elaborate orchestration to protect itself against a harmful disorder. Such insight into amyloid and autoimmunity may offer a novel therapeutic approach to regenerative medicine for neurodegenerative diseases, diabetes, and arthritis.

## Author Contributions

Y-MH and H-LZ contributed to the conception and design of the study. Y-MH wrote the first draft of the manuscript. X-ZH, JS, L-JG, Y-HP, and WL performed the study research. Y-MH, X-ZH, and H-LZ revised the manuscript for the final version. Y-MH is the guarantor of this work and takes responsibility for the integrity of the data and the accuracy of the data analysis. All authors read and approved the submitted version.

### Conflict of Interest

The authors declare that the research was conducted in the absence of any commercial or financial relationships that could be construed as a potential conflict of interest.
